# Accelerating malaria elimination in Cambodia: an intensified approach for targeting at-risk populations

**DOI:** 10.1186/s12936-022-04234-2

**Published:** 2022-07-02

**Authors:** Siv Sovannaroth, Pengby Ngor, Vichka Khy, Julia C. Dunn, Michelle K. Burbach, Sovann Peng, Sarath Mak, Krung Siv, Giulia Manzoni, Jean Olivier Guintran, Luciano Tuseo, Rekol Huy

**Affiliations:** 1 National Center for Parasitology, Entomology and Malaria Control (CNM), Phnom Penh, Cambodia; 2 Clinton Health Access Initiative, Phnom Penh, Cambodia; 3 Catholic Relief Services, Phnom Penh, Cambodia; 4 PSI, Phnom Penh, Cambodia; 5 USAID/PMI/URC, Phnom Penh, Cambodia; 6 World Health Organization, Phnom Penh, Cambodia

## Abstract

**Background:**

Malaria in Cambodia has decreased by 90.8% between 2010 and 2020, driven by the commitment of the National Center for Parasitology, Entomology and Malaria (CNM) and the achievements of the roll-out of a village malaria worker programme. However, in the first seven months of 2018, CNM identified a 207% increase (11,969 to 36,778) in confirmed malaria cases compared to the same months in the previous year. To address this increase, CNM developed the “Intensification Plan” (IP), implemented between October 2018 and December 2020.

**Methods:**

The structure of the IP was summarized, including the selection of sites, the interventions implemented in the selected health facility catchment areas (HFCAs) and the monitoring and evaluation process. Data on IP interventions were collected by CNM and civil society organisations. Data on malaria cases and tests from all HFCAs in Cambodia from January 2018 to December 2020 were sourced from the Cambodia Malaria Information System (MIS) and WHO Malaria Elimination Database. Malaria data from IP HFCAs and non-IP HFCAs was analysed and compared to present the changes in malaria testing and confirmed cases before and during implementation of the IP.

**Results:**

Between October 2018 and December 2020, through the IP 16,902 forest packs and 293,090 long-lasting insecticide treated nets were distributed. In the 45 HFCAs included in the IP, 431,143 malaria tests were performed and 29,819 malaria cases were diagnosed, 5364 (18%) of which were *Plasmodium falciparum*/mixed cases. During the intervention period, over all HFCAs included in IP, *P. falciparum*/mixed cases declined from 1029 to 39, a 96.2% decrease, and from 25.4 *P. falciparum*/mixed cases per HFCA to 0.9. HFCAs not included in IP declined from 468 to 43 cases, a 90.8% decrease, showing that routine malaria activities in Cambodia were also playing an important contribution to malaria control.

**Conclusions:**

Over the course of IP implementation there was a substantial increase in malaria testing and both overall malaria cases and *P. falciparum*/mixed cases decreased month on month. The initiative yields lessons learned for Cambodia to reach the final stage of elimination as well as for other countries aiming to accelerate their malaria control programmes.

**Supplementary Information:**

The online version contains supplementary material available at 10.1186/s12936-022-04234-2.

## Background

Cambodia has made significant progress in malaria control over the last decade. Confirmed malaria cases declined from 106,228 to 9771 cases between 2010 and 2020, a 90.8% decrease. Cambodia still accounted for 13.4% of cases in the Southeast Asia region in 2020 [1, 2], and has received particular attention from the global malaria community since artemisinin resistance was confirmed in 2008 [3], requiring continued vigilance and rapid malaria control to ensure resistance does not derail Cambodia’s elimination efforts or spread to other global regions. In 2011, the National Strategic Plan for Malaria Elimination (NSP 2011–2025) was signed by the Prime Minister, setting the ambitious goal of achieving malaria elimination in Cambodia by 2025 [4].

In Cambodia, from 2010 to 2014 malaria cases reduced by 47% [1] and malaria-related deaths reduced by 88.1% (151 to 18) [5]. This decline was attributed to distribution of insecticide-treated nets (ITNs) as well as the high number of tests performed and cases detected through the village malaria worker (VMW) programme, introduced in 2004 [6]. As malaria cases have decreased in Cambodia, infection has become increasingly focal in hotspots across the country and in populations that are routinely harder to reach and further from points of care [3, 7, 8]. According to the 2013 Cambodia Malaria Indicator Survey, forest-goers (people who work and/or sleep in the forest) and those who travelled had higher odds of malaria infection diagnosed through PCR (odds ratio of 5.8 and 2.3, respectively) [9]. Forests represent a hot spot for malaria transmission and, therefore, mobile and migrant populations involved in forest activities are at high risk of contracting the disease [10].

The VMW programme was suspended from 2014 to 2017. The years following 2014 saw a substantial increase in malaria cases, with a particularly large increase of 102% between 2016 and 2017 [11]. Following the reinstatement of the VMW programme, this increase continued into 2018, culminating in a 207% increase (11,969 to 36,778) in cases January–July 2018 compared to 2017 [11]. This was likely facilitated by a 35% increase in testing (213,585 to 289,325) between 2017 and 2018 [12]. The Malaria Elimination Action Framework (MEAF) 2016–2020 [1], written by the National Center for Parasitology, Entomology and Malaria (CNM), highlighted the implementation of aggressive approaches to reduce malaria in high-risk populations. Informed by the MEAF, to combat this high level of cases, CNM, the Ministry of Health (MoH) and the World Health Organization (WHO) determined that an intensive response was required that targeted hotspots of increased malaria transmission. This plan eventually came to be known as the “Intensification Plan” (IP). The IP took place from October 2018 to December 2020 in 45 health facility catchment areas (HFCAs) across Cambodia.

In this paper, the methods of the Intensification Plan, including objectives, sites, interventions, and M&E through routine data collection and monthly data reviews are presented. Both routine malaria surveillance data and data collected specifically for the IP are used to present the achievements of the IP and analyse change in malaria cases, with a focus on *Plasmodium falciparum*/mixed infections, both in the IP HFCAs themselves and in comparison to the rest of the country.

## Methods

### Objectives of the Intensification Plan

The IP had two objectives aimed at reducing transmission in the areas of the country with the highest malaria burden: (1) improving programme coordination to ensure full implementation of the country’s Malaria Elimination Action Framework (MEAF) (2016–2020) and (2) implementing aggressive approaches to deploy interventions that would rapidly reduce the parasite reservoir among high-risk populations. The IP focused on reaching forest goers and migrant and mobile populations (MMPs) who may enter the forest for logging or other economic purposes and can stay in the forest for up to 2 weeks on each trip. The first phase of IP (IP1) took place from October 2018 to October 2019. The second phase of IP (IP2) took place from November 2019 to December 2020.

### Sites

**Fig. 1:**
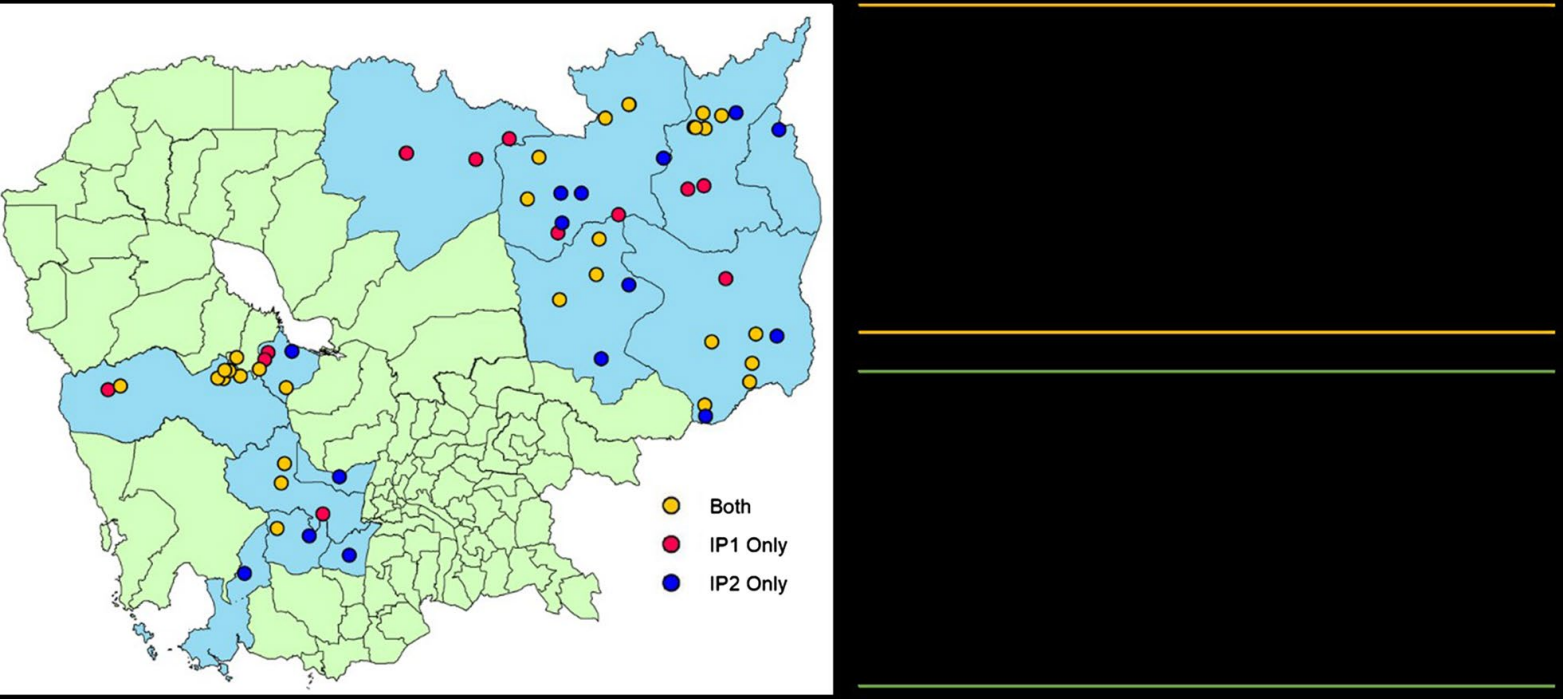
Selected sites to receive Intensification Plan interventions and number of mobile malaria workers (MMWs) assigned based on identified malaria hotspots

To determine the geographical area of intervention, CNM and partners used MIS data to identify provinces, operational districts (ODs) and villages with the highest reported burden of malaria cases. IP1, seven provinces and nine ODs were selected (Fig. 1). In the selected ODs, the 30 highest burden HFCAs were chosen to be included in IP. These 30 HFCAs accounted for 75% of all malaria cases in the country in 2018 and 2019. Sites were reselected at the beginning of IP2; six out of seven provinces remained the same, with one change replacing Preah Vihear for Preah Sihanouk given improvement in the situation in Preah Vihear. 12 ODs with the highest *P. falciparum* cases (as opposed to malaria cases of any species) were selected and 36 HFCAs within these 12 ODs. These HFCAs represented 77% of *P. falciparum* cases in the country from January 2018 to June 2019 and was deemed the maximum number of HFCAs that was programmatically feasible to run and manage the intensified activities. Overall, 45 HFCAs were included over the duration of the IP; 21 HFCAs in both IP1 and IP2, 9 HFCAs in IP1 only and 15 HFCAs in IP2 only. The full list of HFCAs included in IP are provided in Additional file 1: Table S1. CNM used the MIS data and consultations with each HFCA to identify “village hotspots”, areas where at-risk populations of forest-goers resided or transited through. These 141 hotspots (Additional file 1: Table S1) became the focus geography sites for the IP to conduct interventions. Each site received technical support from CNM, the World Health Organization (WHO), the Clinton Health Access Initiative (CHAI), and from a Civil Society Organization based in the corresponding geographic area, namely Catholic Relief Services, CARE, Population Services International (PSI), University Research Co. (URC).

### Interventions

Under the primary objective of the IP, implementing ODs were provided additional support from CNM, WHO and CSOs to improve programme coordination and ensure the effective implementation of case management, including high levels of malaria testing, complete treatment for all those diagnosed with malaria, and effective referral for any severe cases. At the beginning of each phase of IP, long-lasting insecticide treated nets (LLINs) were distributed to any households in the target villages that did not have enough (less than one net per 1.8 people). Additional LLINs were then available for continuous distribution over the course of the IP. The IP included additional technical support and supervision from CNM to ODs, verifying optimal coverage of LLINs in the villages with high incidence and ensuring full attendance at VMW monthly meetings. VMWs conducted monthly meetings to set testing targets and refill case management supplies. ODs were encouraged to attend the meetings to review data and coach on performance.

The second objective of the IP was to implement aggressive approaches to target high-risk populations and hasten the decline of *P. falciparum* cases in the target sites. The main intervention was hiring additional MMWs that were targeted to IP sites and identified hotspots. MMWs were based closer to forested areas where MMPs usually travel and conducted specialized activities focused on forest workers and mobile migrant populations. Performance of MMWs was continuously tracked through monthly VMW/MMW meetings. MMWs also attended several trainings over the course of IP to ensure their knowledge on testing and treating of malaria was up to date and to expand their toolkit (e.g. adding paracetamol and mebendazole to treat those testing negative for malaria). There were also a certain number of MMWs within the targeted sites that were not managed through CNM and the IP, but through the Malaria Consortium [13]. In the following analyses these MMWs are deemed MMW (Not IP).

Responsibilities of MMW included the following:

1. Test: Perform rapid diagnostic tests (RDTs) on all suspected cases according to CNM’s criteria, namely anyone with fever or who had travelled to the forest in the last 1 week.
2. Treat: Provide anti-malarial treatment according to national guidelines, including single low dose primaquine (SLDP) for all *P. falciparum*/mix cases in eligible individuals [14]. The IP expanded access to SLDP for non-pregnant, non-breastfeeding individuals weighing 20 kg and above, where previously the weight requirement was 50 kg, and actively followed-up with MMWs to ensure all *P. falciparum*/mix cases had received SLDP.
3. Track: Keep complete records of all activities including patient consultations on case reporting forms, active case detection (ACD) activities, questionnaires, and forest pack registries.
4. Refer: Refer severe cases to health facilities immediately.
5. Malaria knowledge: Attend trainings and routine monthly meetings for VMW/MMW to ensure good knowledge of malaria diagnosis and treatment.
6. *Work over extended hours* especially when forest-goers are active, this means being accessible 24 h if a patient requests a service.
7. Active test and treat: Twice a month, travel to malaria hot spots in the forest to conduct ACD.
8. Commodity supply: Keep adequate stocks of RDTs and malaria drugs; attend monthly meetings to report stock status, provide paper reports, and replenish stocks.
9. Forest pack distribution: Distribute forest packs to target populations. Forest packs included a backpack, information, education and communication/behavior change communication (IEC/BCC) materials, a hammock net, and (added in IP2) insect repellent. Insect repellent top-ups were also available. The first forest pack distribution took place in May 2019.
10. Perform IEC/BCC activities: Lead information sessions to educate the community about malaria signs and symptoms, provide health education to patients during consultations, play loudspeaker recordings regularly and display educational posters.
11. *Offering optional products for negative malaria cases*, such as paracetamol for fever reduction and mebendazole for de-worming according to national treatment guidelines (IP2 only).
12. Identifying co-travellers: any MMP that testing positive was asked by the MMW for the contact information of their co-travellers. The co-travellers were invited for testing and were provided malaria prevention messages (IP2 only).

For MMWs to perform their responsibilities effectively, the supply chain for RDTs, artemisinin combination therapies (ACTs) (artesunate-mefloquine [ASMQ] and primaquine) and forest packs needed to be improved. At central level, CNM and key partners such as UNOPS, WHO and the implementing CSOs joined together to conduct monthly supply chain meetings to ensure the pipeline of supplies was being accurately forecasted, ordered and distributed timely to subnational levels, in coordination with the Central Medical Stored and subnational partners. The working group also monitored corresponding supplies that MMW need such as weight scales, thermometers, gloves and uniforms for MMW to identify themselves (in IP2).

In non-IP sites, the national surveillance and case management guidelines were followed. Briefly, standard of care was RDT or microscopy testing of all suspected cases, ACT treatment for all cases (*P. falciparum*, *Plasmodium vivax* and mixed infections), SLDP for *P. falciparum*/mix cases, and referral to a hospital for any severe cases. VMWs were situated in the highest burden villages, informed by the national stratification (3025 in 2018, 3376 in 2019, 3675 in 2020). All malaria cases from HCs were entered into the MIS at the time of diagnosis and all cases from VMWs were entered on a monthly basis, after the monthly VMW meetings which also served as regular supervision of VMWs.

For the majority of the IP period, radical cure was not available in Cambodia and *P. vivax* cases were treated with ACT. From November 2019 to December 2020, radical cure was piloted in four provinces, one of which (Kampong Speu) was also included in IP. Over the pilot, any adult male *P. vivax* cases were referred to the nearest HC and tested for G6PD deficiency. If they were G6PD normal they were treated with 14-day primaquine. All other cases were treated with ACT.

### Monitoring and evaluation

Data on malaria testing and cases was routinely entered into the data management system by health centres (HCs), VMWs and MMWs. Each partner involved in IP also collected malaria testing and case data, disaggregated by cadre, in a “CSO Scorecard” to send to CNM and verify against their data, as well as data on IP interventions such as number of forest packs distributed, number of ACD visits and attendance at VMW/MMW monthly meetings. MIS data was compared to CSO Scorecard data and any errors or discrepancies (for example—missing data, more cases than tests, incorrect summation of malaria species to total cases) were clarified with the CSO and corrected in the relevant database. Central CNM staff, with support of WHO and CHAI, analysed the malaria data on a monthly basis (including mapping case data to track any changes in malaria epidemiology). CNM led partner meetings for problem solving and decision making. These “Data Review and Action Meetings” had the goal to provide consistent data review, providing feedback to CSOs and facilitating timely action such as responding to stock outs and flagging HCs/MMWs not performing the target number of outreach visits.

### Data analysis

The timeline for data analysis covers pre-IP (January 2018–September 2018), IP1 (October 2018–October 2019) and IP2 (November 2019–December 2020). Data were collated from the CSO Scorecards, the Cambodia Malaria Information System (MIS) and from the WHO Malaria Elimination Database (MEDB) for all HFCAs in Cambodia. This includes IP intervention data (number of MMWs, number of MMW outreach visits, forest packs distributed, LLINs distributed, repellents distributed) and malaria epidemiology data (tests, treatments, cases). Information on forest packs was collected throughout the IP using “MMW Forest Pack questionnaires”. In March 2020, a review of these questionnaires was conducted, providing further information on forest pack distribution during IP.

To analyse whether the IP had been a factor in driving a decline in malaria cases in the implementing HFCAs, the change in *P. falciparum*/mix cases was compared before and during IP. For this analysis, HFCAs were treated as an IP HFCA if they had been included in any phase of IP (n = 45). Firstly, a segmented interrupted time series was carried out by fitting separate Poisson regression models (log link) to *P. falciparum*/mix cases in (1) Non-IP HFCAs, (2) IP HFCAs (pre-IP rollout) and (3) IP HFCAs (post-IP rollout). The counterfactual trend for IP HFCAs was extrapolated with the pre-IP intercept and gradient parameters. Secondly, a controlled interrupted time series analysis was carried out by fitting a Poisson regression model to *P. falciparum*/mix cases in IP HFCAs with fixed terms for month (to account for seasonality), *P. falciparum*/mix cases in non-IP HFCAs (to control for decline outside of IP) and timepoint interacting with IP Phase (pre/post IP rollout). To account for autocorrelation, standard errors and confidence intervals were calculated with the Newey-West method with a lag of 1. A formula published by Altman and Bland [15] was used to calculate the statistical significance of the difference between rate ratios. Data analysis was completed and figures prepared using RStudio (v 4.0.2, Vienna, Austria).

## Results

### Reporting

To track the deployment of IP interventions and assess epidemiological impact, data was reported to by all health cadres. If monthly data had not been submitted, they were actively followed up by central CNM staff and the responsible CSO. As such, reporting completeness from HCs was 100% over the course of the IP. Data from VMWs and MMWs were collated during monthly meetings. These meetings were tracked via IP surveillance. 788 VMW/MMW meetings out of a possible 893 (88.2%) took place over the course of the IP, attendance was 90.4%. Reporting completeness from VMWs and MMWs was lower than HCs, at 82.2% over the course of IP. There was a drop-off in reporting completeness from VMWs and MMWs between IP1 and IP2, from 96 to 84%.

### Intensification Plan interventions

To reinforce the implementation of the interventions, 141 MMWs (one per village) were recruited over the IP, with an average of 8.8 MMWs active per OD. Over the course of the IP 16,902 forest packs and 293,090 LLINs were distributed (Table 1). The forest-pack survey shows that between May 2019 (when forest packs were distributed) and March 2020 78.7% of MMPs sleeping overnight in the forest had received a forest pack from a MMW; 71.8% of eligible *P. falciparum*/mix cases received SLDP, increasing from 65.4% in IP1 to 86.7% in IP2. The proportion of correctly treated *P. falciparum*/mix infections was variable across provinces; from 80.7% in Pursat to 62.1% in Stung Treng.

**Table 1:** Malaria cases and interventions over the intensification plan (IP), stratified by IP phase and province

	n HFs	Total Tests	Total Cases	Pf/Mix Cases (% of total cases)	SLDP Treatments given (eligible cases only*)	Number of active MMWs	MMW Outreach Visits	Forest Packs distributed	LLINs distributed	Repellents distributed
Total	45	431,143	29,819	5817 (19.5)	2785	141	5228	16,902	293,090	7670
*IP Phase*										
IP1 (Oct 2018-Oct 2019)	30	142,927	21,598	4725 (21.9)	1776	107	1914	11,089	29,568	0
IP2 (Nov 2019-Dec 2020)	36	288,216	8221	1092 (13.3)	1009	141	3314	5813	263,522	7670**
*Province*										
Kampong Speu	7	52,141	6385	1746 (27.3)	1084	18	510	2384	55,108	367
Kratie	4	42,385	1346	376 (27.9)	134	12	382	1317	27,379	967
Mondul Kiri	8	55,944	5015	1192 (23,8)	454	23	706	2405	5462	1175
Preah Sihanouk	1	797	15	0	0	1	3	30	4771	32
Preah Vihear	2	7231	1023	59 (5.8)	6	6	100	1878	528	0
Pursat	8	88,096	8850	1136 (12.8)	535	48	2083	5358	100,967	2913
Ratanakiri	6	64,102	2932	562 (19.2)	277	16	535	2448	12,182	734
Stung Treng	9	120,447	4253	746 (17.5)	295	23	909	1082	86,693	1482

HF: health facility; Pf/mix: *Plasmodium falciparum*/mixed; SLDP: single low-dose primaquine; MMW: mobile malaria worker; LLIN: long-lasting insecticide treated net

*Patients were eligible for SLDP treatment if they weighed over or equal to 20 kg, weren’t pregnant and weren’t breastfeeding. Data on Pf/mix cases eligible for treatment shows some discrepancies so has not been included

**Insect repellent top-ups were available when MMPs ran out, this is why number of repellents is higher than number of forest packs

**Fig. 2:**
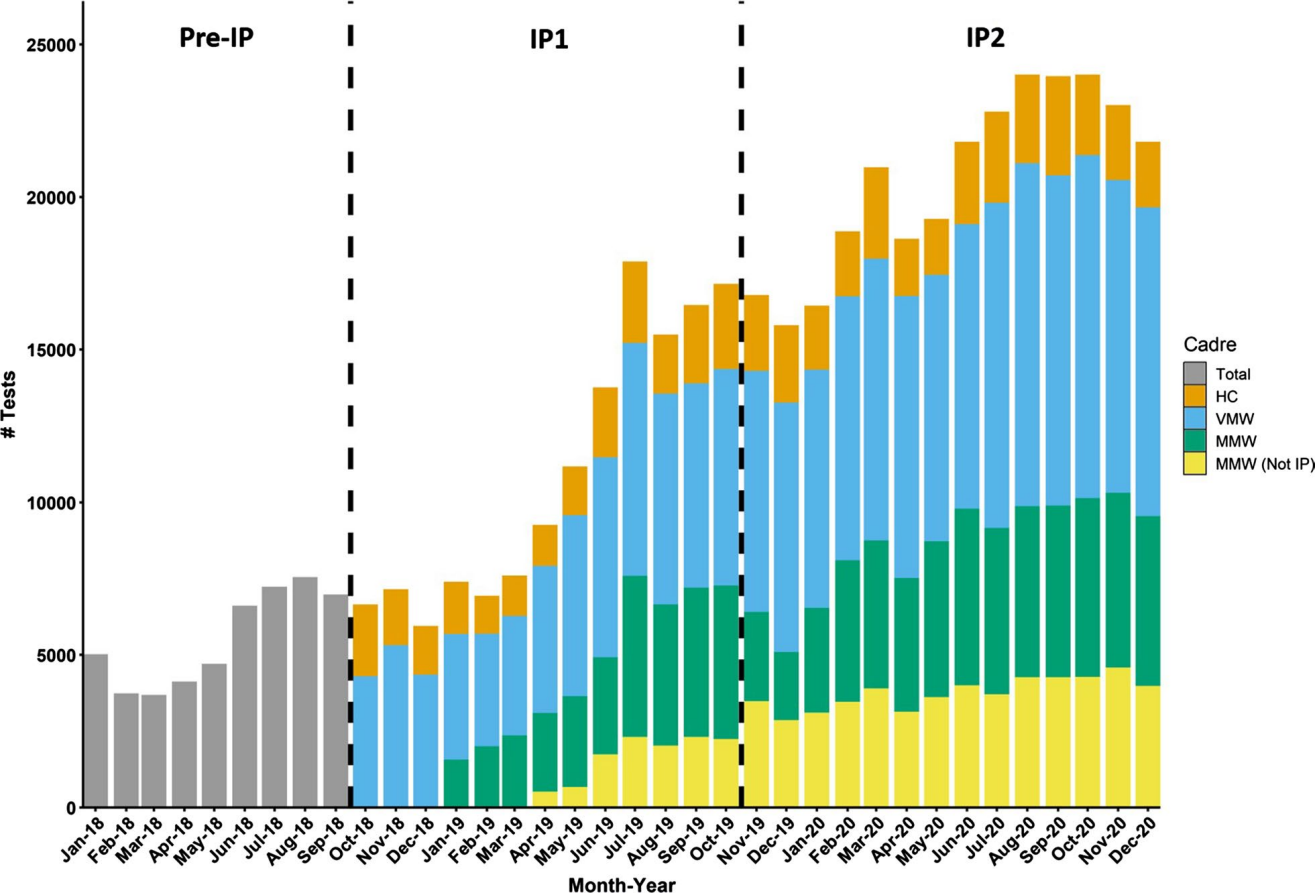
Number of malaria tests performed in intensification plan (IP) health facility catchment areas before and during IP, stratified by health cadre. Total: All tests (cadre disaggregation not available for pre-IP data); HC: health center; MMW (Not IP): mobile malaria workers in IP sites but not managed directly through IP, MMW: mobile malaria workers managed through IP; VMW: volunteer malaria worker. Pre-IP includes IP Phase 1 HFCAs only. IP Phase 1 (30 HFCAs), IP Phase 2 (36 HFCAs)

Over the course of IP 431,143 malaria tests were performed (Table 1), the majority of these tests were performed by VMWs (47.4%) and IP MMWs (23.4%) (Fig. 2). Between the two phases of IP, testing increased by 101.6% with average tests per HC per month increasing from 366.5 to 571.9. HCs had the highest positivity rate at 16.2%, followed by VMWs (7.2%), MMWs (3.7%) and MMWs not hired through IP (2.4%). Of the total tests conducted during IP, 81,462 (18.9%) were during ACD activities with a test positivity rate (TPR) of 2.5%.

### Malaria epidemiology

**Fig. 3:**
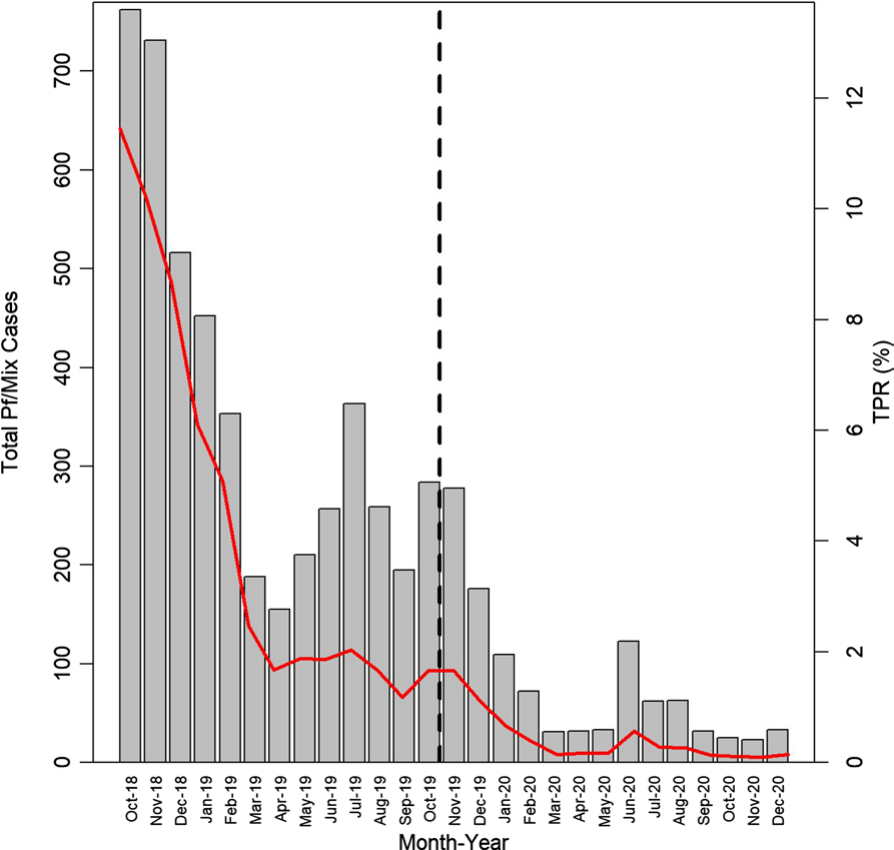
Number of *Plasmodium falciparum*/mix malaria cases (grey bars) and test positivity rate (TPR—red solid line) in intensification plan (IP) health facility catchment areas (HFCAs) over the course of the IP. Left of black dotted line: IP Phase 1 (30 HFCAs); right of black dotted line: IP Phase 2 (36 HFCAs)

Over IP there were 29,819 malaria cases diagnosed, 5817 (19.5%) of which were *P. falciparum*/mix cases (Fig. 3). Between IP1 and IP2 *P. falciparum*/mix cases decreased by 76.9% (4725 to 1092) and from 12.1 cases per HFCA per month to 2.2. In the 30 IP1 HFCAs, *P. falciparum*/mix cases decreased from 762 to 284 (62.7%) from October 2018 to October 2019. In the 36 IP2 HFCAs cases decreased from 278 to 23 (91.7%) from November 2019 to November 2020.

**Fig. 4:**
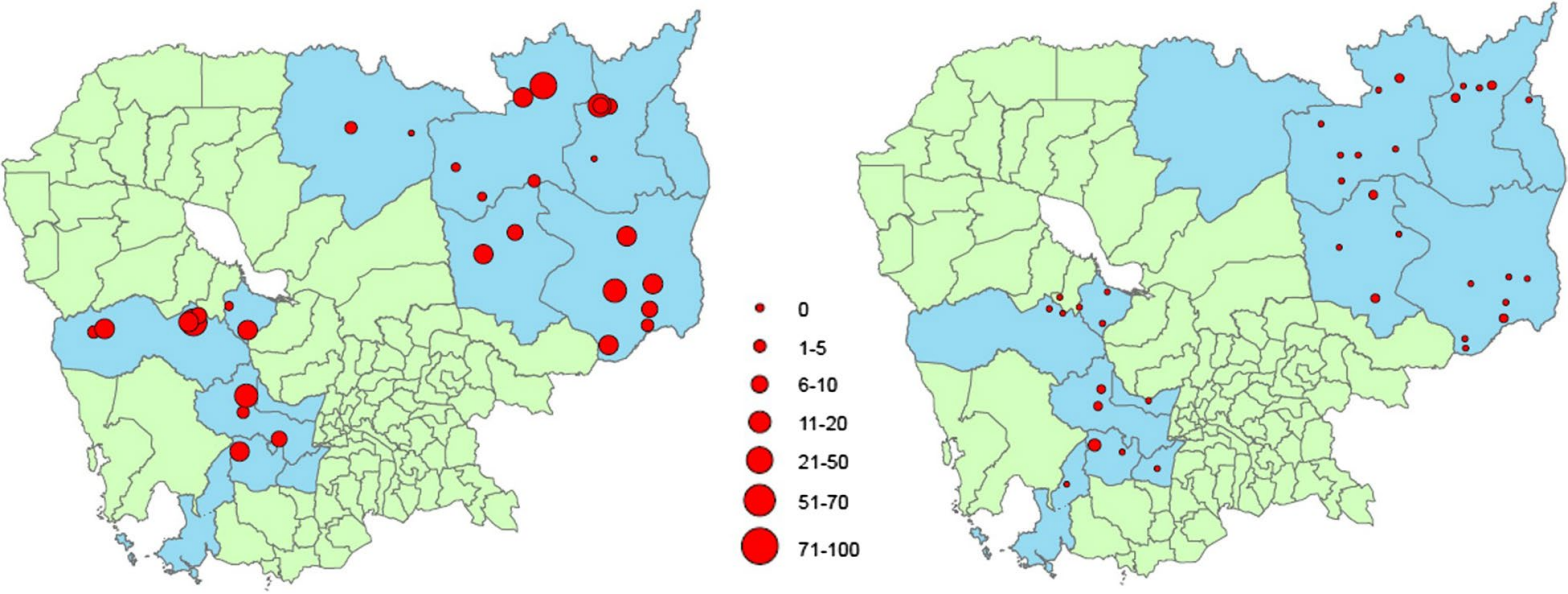
Number of *Plasmodium falciparum*/mix malaria cases (red dots) in intensification plan (IP) health facility catchment areas (HFCAs). Left) Beginning of IP (IP1 HFCAs)—October 2018 and Right) End of IP (IP2 HFCAs)—October 2020. Blue polygons: IP operational districts; green polygons: non-IP operational districts

*Plasmodium falciparum*/mix cases decreased on a HFCA level as well as overall (Fig. 4). In the first three months of IP1 there were 63.5 *P. falciparum*/mix cases per IP1 HFCA. This declined to 23.4 in the last three months of IP1 with 15 HFCAs with zero *P. falciparum*/mix cases in this time. Over IP2, there were 14.8 *P. falciparum*/mix cases per IP2 HFCA in the first three months of IP2, dropping to 2.25 in the last three months of IP2 and 19 HFCAs with zero *P. falciparum*/mix cases in those three months.

On an OD level, the annual parasite incidence per 1000 people (API) in 2018 was 7.7 in IP ODs and 0.3 in non-IP ODs. By 2020 this had decreased to 0.4 in IP ODs (94.7%) and 0.01 in non-IP ODs (96.4%). Non-IP ODs in the same province as IP ODs saw a greater decrease in API than ODs in other provinces (99.5% and 95.9%, respectively), suggesting a spillover effect of the IP interventions into neighbouring areas, possibly due to the IP interventions targeting MMPs, such as forest-goers, that may spread malaria beyond high transmission areas.

**Fig. 5:**
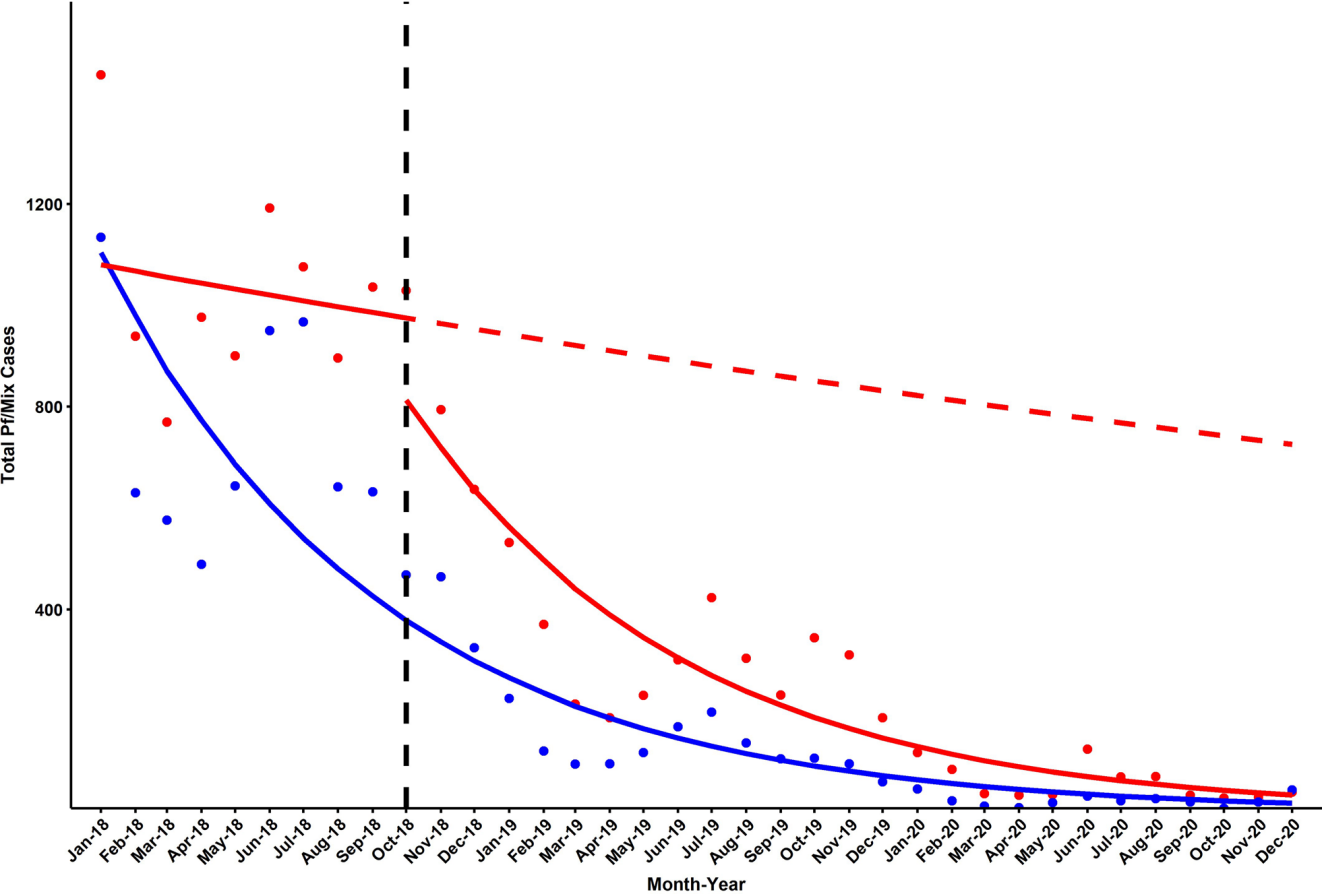
Cumulative number of *P. falciparum*/mix cases in all health facility catchment areas (HFCAs) in Cambodia. Black dashed line: rollout date of IP. Red: IP HFCAs (n = 45); Blue: Non-IP HFCAs (n = 897). Red dashed line: extrapolated counterfactual from pre-IP model. HFCAs are classified as an IP HFCA if they were included in either/both of Phase 1 or Phase 2

The IP was targeted at the HFCAs with the highest burden of *P. falciparum*/mix cases in Cambodia in an attempt to hasten the reduction of cases and achieve elimination. Figure 5 shows the cumulative *P. falciparum*/mix cases from all HFCAs in Cambodia, stratified by whether they are non-IP HFCAs (n = 897) or IP HFCAs (irrespective of IP phase, n = 45). The change in cases over the IP periods (pre and during IP) were quantified by rate ratios (RR). From a controlled interrupted time series analysis, incorporating non-IP HFCAs as a control and adjusting for seasonality, the RR pre-IP was 0.90 (95% CI 0.86–0.93, *p* < 0.0001) and during IP was 0.88 (95% CI 0.86–0.89, *p* < 0.0001). The difference between the two RRs is not statistically significant (*p* = 0.3). This indicates that, even for controlling for the decline in areas where IP was not carried out, IP areas were experiencing a decline in malaria cases. There is evidence for an acceleration in decline, a reduction in the RR, post-IP but the difference is not statistically significant.

## Discussion

While malaria cases increased by more than 100% from 2016 to 2017, over the course of the Intensification Plan (October 2018 to December 2020) there was a 91.6% decline in malaria cases nationwide [11] with a 95.7% decline in IP HFCAs alone. Whilst causality between IP interventions and the change in malaria cases cannot be guaranteed, there has been a substantial decline in malaria cases in the IP HFCAs. This decline has been concurrent with a decline in non-IP HFCAs. Results from the statistical analysis indicates that there was a reduction in IP areas on top of the reduction seen in non-IP areas. There is evidence that the rate of malaria decline accelerated post rollout of IP, however there is no statistical difference between the RRs pre and during IP. One interpretation of these results is that whilst IP has not caused a reduction that is faster than in non-IP HFCAs, it has brought the IP HFCAs in line with the already impressive reduction in malaria cases in the rest of the country. Areas that were once struggling with malaria control have received the added boost they required to be in line with national malaria decline. *P. falciparum*/mix cases remain clustered in mostly the same HFCAs as when IP began, 63.2% of reported *P. falciparum*/mix cases in 2021, were in HFCAs included in IP. However, the impact of IP in the HFCAs has been sustained after it ended; of the 45 HFCAs included in IP, 17 reported zero *P. falciparum*/mix cases in 2021 and a further 17 have reported less than five *P. falciparum*/mix cases.

Evidence from both malaria surveillance and research studies indicate that malaria infection is becoming increasingly focalized in specific populations and that onwards transmission is perpetuated by those deemed “hard-to-reach” [16, 17]. The IP took a novel approach to fill this gap by expanding the number of MMWs and targeting their operations towards to the highest burden areas. The increased flexibility of MMWs, both through their base location and through the services they could provide (distributing forest packs, mebendazole and paracetamol) allowed them to access groups of people that were previously missed by HCs and VMWs. Throughout the IP, MMWs contributed 23.4% of tests and 12.7% of malaria cases. Several other operational and research studies have examined the effectiveness of MMW or forest malaria workers (FMWs) in Cambodia, finding similar results that MMWs are able to achieve high testing rates and reach those populations deemed at most risk of malaria infection [13, 18]. It is clear that engagement of the target populations, who may be engaging in illegal forest-based activity, is required to ensure these more mobile workers are accepted and trusted [18].

Whilst the application of new and expanded malaria control interventions was a major advantage of the IP, another focus from CNM was to improve the use of data through monitoring and evaluation. Included in training of HCs, VMWs and MMWs was a particular focus on routine data reporting and how that data would be used to continually monitor performance and rectify issues. The IP initiated a data review and action cycle that brought together CNM and all implementing partners to share and analyse data on a monthly basis. The meeting allowed all parties to share challenges from the field, propose solutions and collectively determining the priorities for the upcoming month. Through this mechanism all partners have transparency, accountability and a shared vision of addressing the most critical and urgent issues. A major achievement of the IP has been to strengthen monitoring, evaluation and response using timely data to ensure interventions are being implemented to reach the forest goer populations in Cambodia. Such a strong focus on M&E resulted in 100% data reporting from the IP health centres.

The IP focused on the reduction of *P. falciparum*/mix malaria cases. Due to the nationwide reduction in these cases, *P. vivax* is now the dominant malaria species in Cambodia, 86.5% of total cases in 2020 [11], and is targeted for elimination by 2025. In this analysis we have focused on *P. falciparum*/mix cases, however, from October 2018 to October 2020 *P. vivax* cases in the 45 IP HFCAs also decreased by 84.9%. Ensuring effective case management, vector control and universal access to care is not malaria species specific. As such, interventions such as those included in the IP are necessary for *P. vivax* elimination as well as *P. falciparum*. Following a pilot in 2019, radical cure for *P. vivax* through primaquine treatment was scaled-up nationwide in December 2020. The strengthening of the VMW programme through programmes such as IP will support other interventions, such as radical cure, through well-trained VMWs and a strong data collection pathway.

The implementation of IP faced several operational challenges. Delays in procurement of repellents meant that the distribution of forest packs happened after May 2019, and repellents were only distributed as part of IP2. While IEC/BCC messages were recorded and distributed on loudspeakers for MMWs to play in the forest, more effort is required to measure the impact of IEC/BCC activities and how they can be better targeted in future programmes. Given the transient nature of MMP and often illegal activities conducted in the forest, it can be difficult to reach the target population with IEC messages. The rainy season in remote areas may have caused data to be delayed in entering into MIS where it was captured in the following month, which can prevent timely analysis and response during these periods. Finally, as with all health programmes, the emergence of the COVID-19 pandemic at the beginning of 2020 impacted the implementation of both routine and IP malaria programmes. For example, in-person meetings with VMWs and MMWs were adapted to COVID-19 measures (e.g. solo instead of group meetings, held in open spaces) and health workers in some areas were reassigned from malaria to COVID activities (e.g. testing and quarantine). CNM applied mitigation measures in accordance with the national guidance on COVID-19 issued by the Royal Government of Cambodia and took steps to limit the exposure of health personnel to COVID-19 including procurement of personal protective equipment and reducing subnational travel [19]. Another limitation of the analysis is the potential impact that COVID-19 may have had on malaria transmission and epidemiology. Cambodia closed its borders in March 2020 and inter-province travel was restricted. This could have impacted malaria transmission due to reduced travel between high-risk areas. However, as there were very few reported COVID cases over the course of 2020 in Cambodia, travel did not seem to be severely impacted and this has not been treated as a significant factor to explain malaria decline. There was also a brief reduction in malaria testing between March and April, however testing remained above pre-pandemic levels. In the analysis presented here how malaria cases changed over the course of the IP, and in comparison to areas not included in IP, has been described. However, causality between IP and decline in malaria cases cannot be guaranteed as other factors, such as climate, have not been included in the analysis.

CNM initially aimed to achieve elimination of *P. falciparum* by 2020, however this has now been extended to 2023 [3]. Valuable lessons were learned from the IP which will be applied to achieve this goal, namely the importance of targeting forest-based populations and the importance of the VMW/MMW cadre. At the end of 2020, CNM, technically supported by WHO, are implementing a programme known as “last mile”, reinforcing foci management activities to accelerate malaria elimination [20, 21]. Based on the vulnerability and receptivity of active foci, additional interventions are implemented. This includes targeted drug administration and intermittent preventive treatment for at-risk populations, as well as weekly fever screening of village-based populations and LLIN/LLIHN distribution. In addition, continuing to support routine malaria case management and surveillance remains essential in achieving elimination. CNM, WHO and CSOs will continue to work to build capacity of health staff at all levels, as well as implementing strong financial management and operational planning to ensure all activities, both routine and one-off, are implemented on time and to the highest standard.

## Conclusion

Through population-targeted activities and routine use of malaria surveillance data *P. falciparum*/mix cases declined by 97.4% (1029 to 27) between October 2018 and October 2020 in the 45 IP HCs. CNM will continue to focus on capacity building for HCs, VMWs and MMWs to determine malaria hotspots as well as fostering collaboration with HCs and local authorities to better track and target MMPs to provide preventive and treatment services. Vector control and raising awareness of malaria prevention and treatment to MMPs and forest goers are continuing priorities for the last mile of malaria elimination in Cambodia.

## Supplementary Information

**Additional file 1: Table S1.** List of selected health centres and villages for Intensification Plan.

## Data Availability

National malaria surveillance data available at https://mis.cnm.gov.kh/. Intensification Plan data available upon reasonable request to the National Center for Parasitology, Entomology and Malaria Control (CNM), Phnom Penh, Cambodia.

## Abbreviations

ACD: Active case detection
ACT: Artemisinin combination therapy
API: Annual parasite incidence
ASMQ: Artesunate-mefloquine
BCC: Behavior change communication
CNM: National Center for Parasitology, Entomology and Malaria
CSO: Civil Society Organization
G6PD: Glucose-6-phosphate Dehydrogenase
GLM: Generalized Linear Model
HC: Health Centre
HFCA: Health facility catchment area
IEC: Information, education and communication
IP: Intensification Plan
IP1: Intensification Plan 1 (October 2018—October 2019)
IP2: Intensification Plan 2 (November 2019–December 2020)
LLIHN: Long-lasting insecticide treated hammock net
LLIN: Long-lasting insecticide treated net
MEAF: Malaria Elimination Action Framework
MIS: Malaria Information System
MMPs: Migrant and mobile populations
MMW: Mobile malaria worker
MoH: Ministry of Health
NSP: National Strategy for Malaria Elimination
OD: Operational district
RDT: Rapid diagnostic tests
SLDP: Single low dose primaquine
TPR: Test positivity rate
VMW: Village malaria worker
WHO: World Health Organization
